# Supplementary material for: Deletion of wfs1 Impairs Oligodendrocyte Precursor Cells Dorsal Distribution and Myelination Through the wfs1-hmgcs1 Axis in Zebrafish
Source: Biology (Basel). 2026 Aug 16;15(16):1402. doi: 10.3390/biology15161402 (PMC13510151; doi:10.3390/biology15161402)
Supplement: Supplementary file 1 [file biology-15-01402-s001.zip › File S1-Transcriptome sequencing data and differentially expressed genes (DEGs) in wfs1-- zebrafish at 5 dpf/2.KEGG/wfs1KOvsWT/ALL/src/dre00260.html]

dre00260


- dre:550524

- Down regulated genes

550524\_1(-2.57926437647488)

- dre:445818

- Up regulated genes

445818(1.93771996696489)

- dre:394103

- Up regulated genes

394103(4.1603195563306)

- Down regulated genes

394103\_1(-2.48669285078694)

- dre:563642

- Up regulated genes

563642(1.74231278020234)

- dre:405800

- Up regulated genes

405800(1.1459925069877)
- dre:619259

- Up regulated genes

619259(1.74407736881054)

- dre:563642

- Up regulated genes

563642(1.74231278020234)

- dre:64607

- Down regulated genes

64607\_1(-2.39285410667541)

- dre:541429

- Up regulated genes

541429(1.54037114709183)

- Down regulated genes

541429\_1(-2.0586358059379)

- dre:563642

- Up regulated genes

563642(1.74231278020234)

- dre:550524

- Down regulated genes

550524\_1(-2.57926437647488)

- dre:541329

- Up regulated genes

541329(9.19981692424844)

- Down regulated genes

541329\_1(-1.63898505916822)

Close
